# New Host-Directed Therapeutics for the Treatment of Clostridioides difficile Infection

**DOI:** 10.1128/mBio.00053-20

**Published:** 2020-03-10

**Authors:** Jourdan A. Andersson, Alex G. Peniche, Cristi L. Galindo, Prapaporn Boonma, Jian Sha, Ruth Ann Luna, Tor C. Savidge, Ashok K. Chopra, Sara M. Dann

**Affiliations:** a Department of Microbiology and Immunology, University of Texas Medical Branch, Galveston, Texas, USA; b Department of Pathology and Immunology, Baylor College of Medicine, Houston, Texas, USA; c Texas Children’s Microbiome Center, Department of Pathology, Texas Children’s Hospital, Houston, Texas, USA; d Department of Internal Medicine, University of Texas Medical Branch, Galveston, Texas, USA; e Department of Cell Biology and Molecular Medicine, Rutgers New Jersey Medical School, Newark, New Jersey, USA; f Faculty of Medicine, King Mongkut’s Institute of Technology Ladkrabang, Bangkok, Thailand; g Institute for Human Infections and Immunity, University of Texas Medical Branch, Galveston, Texas, USA; h Institute for Translational Sciences, University of Texas Medical Branch, Galveston, Texas, USA; McGovern Medical School

**Keywords:** *Clostridioides difficile*, germfree mice, host-directed therapeutics, mechanism of action, microbiota, mouse model of infection

## Abstract

Frequent and excessive use of antibiotics primes patients to Clostridioides difficile infection (CDI), which leads to fatal pseudomembranous colitis, with limited treatment options. In earlier reports, we used a drug repurposing strategy and identified amoxapine (an antidepressant), doxapram (a breathing stimulant), and trifluoperazine (an antipsychotic), which provided significant protection to mice against lethal infections with several pathogens, including C. difficile. However, the mechanisms of action of these drugs were not known. Here, we provide evidence that all three drugs offered protection against experimental CDI by reducing bacterial burden and toxin levels, although the drugs were neither bacteriostatic nor bactericidal in nature and had minimal impact on the composition of the microbiota. Drug-mediated protection was dependent on the presence of the microbiota, implicating its role in evoking host defenses that promoted protective immunity. By utilizing transcriptome sequencing (RNA-seq), we identified that each drug increased expression of several innate immune response-related genes, including those involved in the recruitment of neutrophils, the production of interleukin 33 (IL-33), and the IL-22 signaling pathway. The RNA-seq data on selected genes were confirmed by quantitative real-time PCR (qRT-PCR) and protein assays. Focusing on amoxapine, which had the best anti-CDI outcome, we demonstrated that neutralization of IL-33 or depletion of neutrophils resulted in loss of drug efficacy. Overall, our lead drugs promote disease alleviation and survival in the murine model through activation of IL-33 and by clearing the pathogen through host defense mechanisms that critically include an early influx of neutrophils.

## Abstract (precis)

Clostridioides difficile is a spore-forming anaerobic bacterium and the leading cause of antibiotic-associated colitis. With few therapeutic options and high rates of disease recurrence, the need to develop new treatment options is urgent. Prior studies utilizing a repurposing approach identified three nonantibiotic Food and Drug Administration-approved drugs, amoxapine, doxapram, and trifluoperazine, with efficacy against a broad range of human pathogens; however, the protective mechanisms remained unknown. Here, we identified mechanisms leading to drug efficacy in a murine model of lethal C. difficile infection (CDI), advancing our understanding of the role of these drugs in infectious disease pathogenesis that center on host immune responses to C. difficile. Overall, these studies highlight the crucial involvement of innate immune responses, as well as the importance of immunomodulation as a potential therapeutic option to combat CDI.

## INTRODUCTION

Clostridioides difficile infection (CDI) is the most serious cause of antibiotic-associated diarrhea, which can quickly progress to fatal disease if not promptly treated. Therapy for CDI is challenging, and infections are most common in hospitalized patients, typically aged 65 or older, already rendered vulnerable to infection due to comorbid medical conditions (1–3). Even with effective therapy, recurrence rates of CDI are high. Within 30 days of completing a standard course of antibiotics for an initial episode, ∼15 to 30% of patients will develop a recurrent infection and, of these, up to 60% will experience additional relapses (4, 5). In addition to being debilitating and lowering patients’ quality of life, frequent recurrences are associated with increased mortality and higher health care costs (6, 7).

There are few therapeutic options available for treating CDI. Current guidelines recommend treating initial and recurrent infections, even those that are mild, with vancomycin or fidaxomicin (8). Although vancomycin is effective for most cases, C. difficile isolates with resistance or reduced susceptibility to the antibiotic have emerged worldwide (9–11). If these rates were to increase or if mutations leading to decreased susceptibility to fidaxomicin were to develop, health care providers would be faced with a serious challenge. Further, the effectiveness of antibiotic therapy declines with each recurrence, leaving fecal microbiota transplantation (FMT) as a last option for patients with treatment failure. While FMT has shown great promise in combating recurrent CDI (12–14), it is not Food and Drug Administration (FDA) approved and is associated with a variety of risks, including lack of knowledge of long-term health effects and the transfer of potentially fatal multidrug-resistant organisms to recipients, as was recently reported (15).

Despite advances in technology and medical knowledge, the traditional process of drug discovery has resulted in few new classes of FDA-approved antibiotics over the last several decades (16, 17). Major challenges, particularly the escalating costs associated with the length of time required for development and meeting regulatory requirements, have decreased investors’ interest and support (16, 17). Thus, alternative strategies are needed for discovering and developing therapeutic agents for treating infections that are serious threats to public health.

Drug repurposing or repositioning, a process that involves finding new indications for existing drugs, is one strategy that has proven effective in identifying new treatments for a range of human diseases (18–21). Using this approach, we identified three FDA-approved drugs, amoxapine (AXPN; an antidepressant), doxapram (DXP; a breathing stimulant), and trifluoperazine (TFP; an antipsychotic), which provided protection against fatal pneumonia caused by Yersinia pestis infection (22). None of the drugs possessed antibacterial activity at clinically used doses, suggesting that protection was conferred through host-directed mechanisms (22). Importantly, all three drugs demonstrated broad applicability against a wide range of Gram-negative bacteria, including Klebsiella pneumoniae and Salmonella enterica serovar Typhimurium, and against Gram-positive C. difficile (22, 23). Building upon this work, the present study was designed to evaluate the potential application of AXPN, DXP, and TFP for CDI by elucidating the mechanisms of protection in murine models of infection.

With limited options available to treat CDI, our study provides a new avenue in modulating host innate immune responses as a means to contain infection, with a much-reduced possibility of the bacterium to develop drug resistance or to further alter the microbiota. Since our paper describes the mechanisms associated with the lead drugs in host protection against CDI, the data presented pave the way for rapid preclinical and clinical testing, thus shortening the time to marketing, specifically as the structures, pharmacokinetic/pharmacodynamic properties of the compounds, and safety profiles of these drugs are already known.

## RESULTS

### Lead drugs protect mice from lethal C. difficile infection.

**FIG 1:**
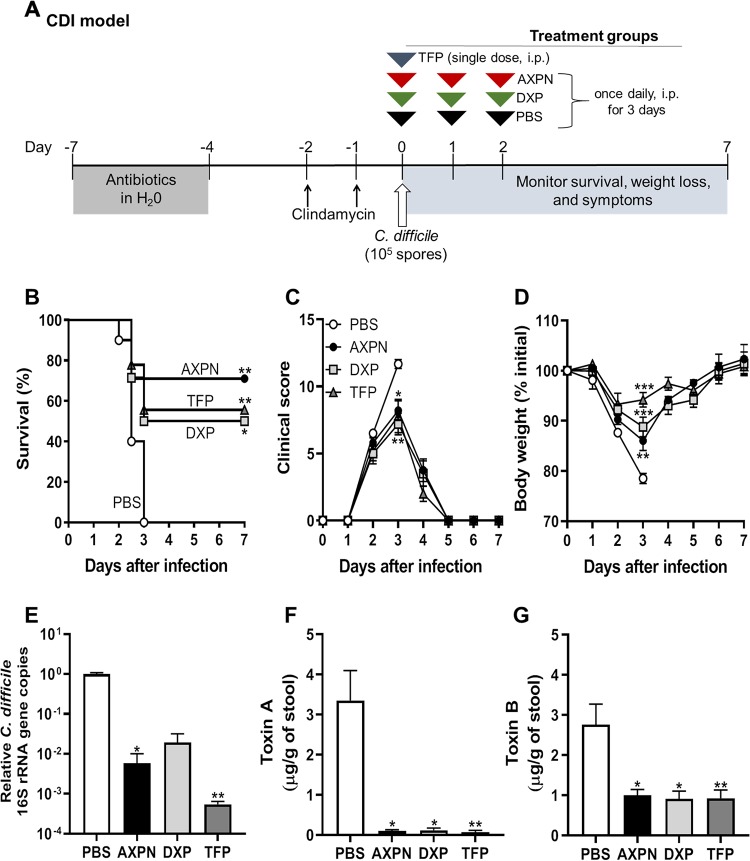
Model of lead drugs protecting against lethal C. difficile infection (CDI). (A) Experimental design for CDI model. C57BL/6 mice were pretreated with antibiotics by administration of an antibiotic cocktail in the drinking water for 3 to 4 days, followed by injections of clindamycin for 1 to 2 days at 24-h intervals. Mice were then orally infected with C. difficile spores and administered lead drugs or the vehicle control PBS, starting at the time of infection. (B) Mice were monitored for survival (*n *= 9 or 10 mice/group). *, *P < *0.05; **, *P < *0.01 (determined by log rank survival statistics). (C and D) Development of clinical symptoms (C) and weight loss (D) were assessed at the indicated time points (mean ± SE, *n *= 9 or 10 mice/time point). *, *P < *0.05; **, *P < *0.01; ***, *P < *0.001, compared to results for PBS controls. (E) At 30 h postinfection, the bacterial burden was determined by qPCR analysis of the quantities of 16S rRNA gene copies of C. difficile relative to the total number of 16S rRNA gene copies in the cecal contents. Expression levels are shown in comparison to that of the PBS-treated group on a log_10_ scale. (F and G) Toxin levels were measured in the cecal contents by ELISA at 30 h postinfection. Data are the mean ± SE of the results of two independent experiments (*n *= 5 to 8 mice/group). *, *P < *0.05; **, *P < *0.01; ***, *P < *0.001, compared to results for the PBS control.

We recently demonstrated in a mouse model of lethal CDI that a combination of a subclinical dose of vancomycin with lead drugs AXPN, DXP, and TFP, administered as adjunct therapy (24 h postinfection), reduced lethality by 80 to 100% (23). While TFP alone provided 60% protection (22), we did not determine the therapeutic efficacy of AXPN and DXP alone. To validate our previous findings, and to evaluate the therapeutic efficacy of each lead drug, adult C57BL/6 mice were pretreated with antibiotics and then administered a lethal dose (10^5^ spores by oral gavage) of C. difficile, followed by drug treatment. As determined in our earlier studies, TFP (1.5 mg/kg, intraperitoneally [i.p.]) was given once at the time of infection, while AXPN (3 mg/kg, i.p.) and DXP (20 mg/kg, i.p.) were administered at 24-h intervals, beginning at the time of infection, for a total of 3 doses (22). The overall experimental design for antibiotic pretreatment, spore challenge, and drug treatment is depicted in Fig. 1A.

By following the outlined treatment protocol (Fig. 1A), all three drugs provided significant protection against lethal infection, with AXPN providing 70%, DXP 50%, and TFP 55% protection (Fig. 1B). In accordance with fulminant disease, drug-treated mice had less severe clinical scores and reduced weight loss, with significant differences noted on day 3 postinfection between drug-treated groups and the vehicle-treated (phosphate-buffered saline [PBS]) control (Fig. 1C and D). Following day 3, which was universally fatal in PBS-treated mice, the surviving drug-treated mice recovered, with clinical scores and weights returning to baseline values (Fig. 1B to D).

To determine if drug efficacy was related to a decrease in bacterial burden or toxin production during infection, we quantified relative C. difficile loads and toxin titers by quantitative PCR (qPCR) and enzyme-linked immunosorbent assay (ELISA), respectively, in cecal contents at 30 h postinfection, a time point prior to when PBS-treated control mice succumbed to infection. AXPN- and TFP-treated mice had significant reductions in C. difficile gene counts, with a pathogen burden up to 3 orders of magnitude lower than that of the PBS-treated control group (Fig. 1E). Although significance was not achieved, a similar trend was observed with DXP treatment. Similarly, C. difficile toxin A and B levels were significantly decreased across all drug treatment groups compared to those of PBS-treated mice (Fig. 1F and G). Taken together, these data indicate that the lead drugs mediate protection by limiting C. difficile growth and toxin production.

### Lead drugs provide protection through host-directed mechanisms.

Having established that each drug alone provides protection against CDI, we sought to elucidate the underlying mechanisms. To determine if the drugs possessed antimicrobial properties, C. difficile was grown in the presence of a 33 μM concentration of each drug (equivalent to 10.35 μg/ml AXPN, 13.69 μg/ml DXP, and 15.58 μg/ml TFP) for 24 h. In comparison to the positive control vancomycin, no drug exhibited bactericidal activity (see Fig. S1A in the supplemental material), with the MIC for each drug being >100 μg/ml (data not shown). With previous reports demonstrating a nanogram therapeutic range in serum (24–27), these results indicate that drug-mediated protection was not due to bacterial killing *in vivo*.

10.1128/mBio.00053-20.1 FIG S1 Impact of lead drugs on C. difficile growth and toxin production. C. difficile VPI 10463 was grown in BHIS in the presence of TFP, AXPN, DXP, or vancomycin (vanc) at a 33 μM concentration or in the presence of the vehicle control PBS. (A) For growth effect, samples were taken at the indicated time points for OD_600_ measurement. (B and C) Toxin production was determined by ELISA in culture supernatant samples taken at 24 h postinoculation. Data represent the mean ± SE of results from two separate experiments, performed in triplicate. ****, *P < *0.0001, compared to results for the vehicle control PBS group. Download FIG S1, TIF file, 0.1 MB. Copyright © 2020 Andersson et al. 2020 Andersson et al. This content is distributed under the terms of the Creative Commons Attribution 4.0 International license.

As toxins A and B are the main virulence factors of C. difficile, we also evaluated whether the drugs directly impact their production. C. difficile was grown in the presence of each drug, as described above, and supernatants were evaluated for toxin levels. Similar to what was observed for bacterial growth, none of the drugs directly affected toxin A or B production (Fig. S1B and C). Collectively, these results indicate that although drug treatment results in decreased bacterial burden and toxin production *in vivo*, protection is not mediated through direct pathogen interaction but rather via indirect effects on the host.

### Lead drugs do not restore a protective microbiome community.

**FIG 2:**
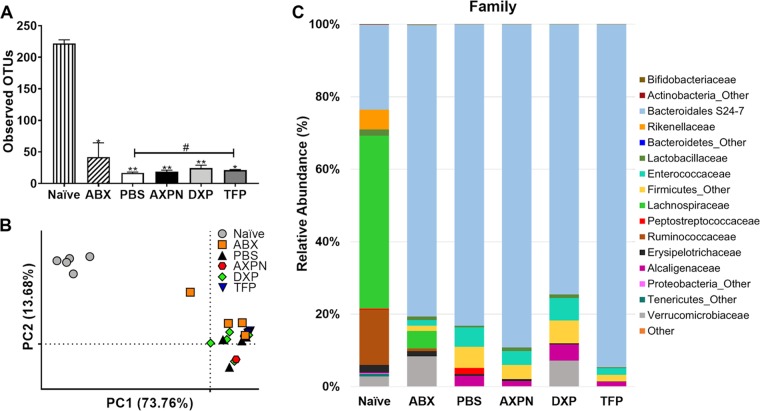
Treatment with lead drugs does not alter the microbiota, which is in a dysbiotic state after antibiotic treatment and CDI. Following antibiotic pretreatment, mice were infected with C. difficile spores and administered lead drugs or the vehicle control PBS (CDI model [Fig. 1A]). Uninfected, but antibiotic-pretreated, mice (ABX) and naive (untouched) mice served as controls. At 30 h postinfection, DNA from the cecal contents of the indicated groups of animals (as described in Table 1) was collected (*n *= 4 or 5 mice/group) and analyzed using the Illumina MiSeq system. (A) Alpha diversity, as measured by observed operational taxonomic units (OTUs). Levels are represented as the mean ± SE. *, *P < *0.05; **, *P < *0.01, compared to results for naive mice, or #, *P < *0.05, between two indicated groups. (B) PCA of weighted UniFrac distances for 16S rRNA V3-V4 sequence data. Each dot corresponds to an individual mouse. (C) Average relative microbial abundance at the family level.

To determine if our lead drugs impacted microbiome community changes induced by antibiotic exposure and CDI in mice, DNA extracted from cecal contents was 16S rRNA gene sequenced on the Illumina MiSeq platform. As described in Table 1, six groups of mice were used for the comparison analysis. A naive group of animals served as a control, which remained untouched, with no antibiotic pretreatment or infection performed. The remaining five groups received antibiotic pretreatment to disrupt the microbiota and render them susceptible to CDI. Of these, four groups were infected with C. difficile spores and given a lead drug (AXPN, DXP, or TFP) or PBS. The final group of mice underwent antibiotic pretreatment but were not infected (ABX) (Table 1). As previously reported (28, 29), antibiotic exposure alone (ABX) led to a significant reduction in microbiome diversity compared to that of naive animals. Thirty hours postinfection, diversity remained limited compared to that of naive mice, regardless of drug treatment (Fig. 2A). Principal-component analysis (PCA) (Fig. 2B), to assess overall compositional differences between individual mice, revealed clear shifts in the compositions of the microbiota following antibiotic pretreatment, which remained similar during infection. As observed for diversity, drug treatment had little impact on microbial composition, which resembled that of the PBS-treated mice in the coordinate space (Fig. 2B). Analysis of dominant phyla revealed expansion of *Bacteroidetes* and reduction of *Firmicutes* following antibiotic pretreatment, which persisted throughout infection and following drug treatments (Fig. 2C). At the family level, drug treatment reduced *Peptostreptococcaceae*, which is reflective of reduced C. difficile burden since it is a family member. No other significant changes were observed among infected groups, indicating that treatment with AXPN, DXP, or TFP did not mediate microbiota community shifts associated with protection against CDI.

**TABLE 1:** Description of groups used in this study

Group	Treatment(s) administered					
	Antibiotic pretreatment	C. difficile infection	PBS treatment	AXPN treatment	DXP treatment	TFP treatment
Naive						
ABX	X					
PBS	X	X	X			
AXPN	X	X		X		
DXP	X	X			X	
TFP	X	X				X

### The microbiota is necessary for lead drug efficacy.

**FIG 3:**
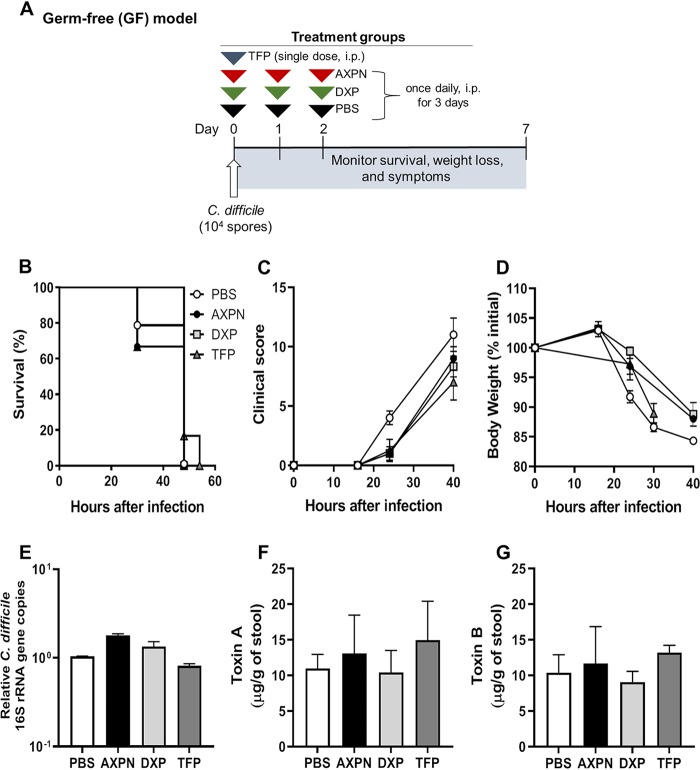
Gut microbiota is required for drug-mediated protection against CDI. (A) Experimental design of CDI infection in germfree (GF) mice. As no antibiotic pretreatment was required, GF mice were infected with C. difficile spores and administered lead drugs or the vehicle control PBS at the time of infection. (B to D) Survival (B), development of clinical scores (C), and weight loss (D) were monitored over the course of infection. (E) Bacterial burden in the cecal contents was determined at the time of necropsy by qPCR analysis of the quantities of 16S rRNA gene copies of C. difficile relative to the total number of 16S rRNA gene copies in the cecal contents. Expression levels are shown in comparison to the PBS-treated group on a log_10_ scale. (F and G) Toxin titers were determined in cecal contents collected at the time of necropsy by toxin ELISA. Data are the mean ± SE of the results for 3 to 6 mice/group.

To determine whether the microbiota contributed to drug-mediated protection against lethal CDI, germfree (GF) mice were infected with a lower challenge dose of C. difficile (10^4^ spores) and administered lead drugs or PBS at the time of infection (Fig. 3A). In sharp contrast to the results with the CDI model (Fig. 1), GF mice showed little improvement in body weight loss or clinical scores with drug treatment, and all succumbed to infection (Fig. 3B to D). There was also no evidence of bacterial clearance in GF mice, with comparable C. difficile burdens and toxin levels across all groups (Fig. 3E to G). These findings support our *in vitro* conclusions that lead drugs do not directly impact C. difficile growth or toxin production but strongly implicate host-microbiome interactions in mediating protection.

### Lead drugs promote induction of cooperative innate host defenses.

**FIG 4:**
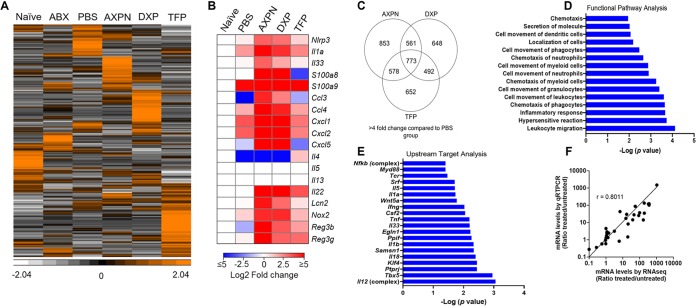
Innate immune defenses against CDI are upregulated following treatment with lead drugs. Following antibiotic pretreatment, mice were infected with C. difficile spores and administered lead drugs or the vehicle control PBS. Uninfected, but antibiotic-pretreated, mice (ABX) and untouched (naive) mice served as controls (Table 1). Thirty hours postinfection, cecal tissue was harvested for total RNA isolation and subjected to RNA-seq analysis. Data represent RNA pooled from 5 mice/group. (A) Hierarchical clustering of 5,894 transcripts representing unique genes altered by >4-fold in one or more groups compared to untreated, naive controls (see Table S1 in the supplemental material). Bright orange represents the highest values (standardized counts per million), white represents the lowest values, and black represents median values. (B) Heat map displaying gene expression differences for select genes of functional interest profiled by RNA-seq. Data shown are log_2_ fold change (treatment groups versus naive controls) in expression plotted using the color scale shown. (C) Venn diagram of 4,557 genes with a 4-fold change in expression induced by drug treatment in comparison to that of the PBS-treated group. (D and E) Enriched pathways of the top upregulated transcripts and upstream targets listed by significance [−log_10_ (*P*)] based on IPA. (F) Correlation between RNA-seq and qRT-PCR expression levels was determined for 20 select genes.

In addition to facilitating colonization resistance to invading pathogens, the intestinal microbiome is a key modulator of host immune defenses (30). To identify which defenses and networks may be associated with drug-mediated protection, we conducted transcriptome sequencing (RNA-seq) analysis to obtain genome-wide expression profiles of the cecal response in the CDI model (Fig. 1A). We chose the cecum as the organ for investigation, since this is the most significantly impacted tissue in murine CDI. Accordingly, we extracted total RNA from whole cecal tissue of mice treated with lead drugs at 30 h postinfection and performed deep sequencing to identify global expression differences among the six groups of controls and treatments (Table 1). As shown by hierarchical clustering, each drug induced a unique expression profile, with 5,894 transcripts altered by >4-fold in one or more groups compared to untreated, naive controls (Fig. 4A; Table S1). As depicted in Fig. 4B, drug treatment promoted or enhanced the induction of many genes encoding products with diverse functions, including calcium-binding proteins with intracellular and extracellular antimicrobial functions (*S100a8* and *S100a9*), chemokines for recruiting neutrophils and monocytes (*Ccl3*, *Ccl4*, *Cxcl1*, *Cxcl2*, *Cxcl5*), proteins involved in the initiation and promotion of immunity and/or inflammation (*Nlrp3*, *Il1a*, *Il33*, and *Il22*), and other factors with antimicrobial activity (*Nos2*, *Lcn2*, *Reg3b*, and *Reg3g*). Of the profiled host transcripts, 4,557 (12.1%) were differentially expressed (fold change > 4) by drug treatment in comparison to the PBS-treated group (Fig. 4C). Expression levels were upregulated for 2,365 (51.9%) genes and downregulated for 2,192 (48.1%) genes. Although each drug induced a unique expression profile, changes in 773 transcripts were shared among the three drugs (Fig. 4C). Analysis of the RNA-seq data using Ingenuity Pathways Analysis (IPA) further revealed enrichment of innate-immune related pathways involved in leukocyte migration, cell movement of granulocytes, phagocytes, and myeloid cells, and chemotaxis of neutrophils (Fig. 4D). Upstream target analysis also revealed enhanced expression of genes targeting several interleukin 1 (IL-1) pathway factors, including myeloid differentiation primary response 88 (*Myd88*), *Il1a*, *Il1b*, *Il18*, and *Il33* (Fig. 4E). To confirm the RNA-seq analysis, mRNA expression levels of 20 select genes were measured by quantitative real-time (qRT)-PCR (Fig. 4F). These included several genes encoding chemokines, including IL-22 and IL-33 (Fig. 4F), which were validated by performing ELISA and multiplex immunoassays to determine protein levels (data not shown).

10.1128/mBio.00053-20.3 TABLE S1 Differentially expressed genes 30 h postinfection. RNA-seq counts for 5,894 genes differentially expressed by >4-fold (increased or decreased) in one or more groups compared to counts for untreated, naive controls. Download Table S1, XLSX file, 0.3 MB. Copyright © 2020 Andersson et al. 2020 Andersson et al. This content is distributed under the terms of the Creative Commons Attribution 4.0 International license.

### AXPN enhancement of innate immune pathways is dependent on the microbiome.

**FIG 5:**
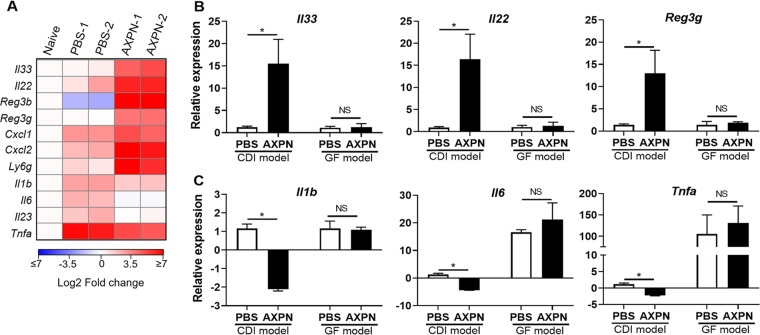
AXPN enhances protective innate immune pathways against CDI and is dependent on the microbiota. (A) Antibiotic-pretreated mice were infected with C. difficile spores and administered AXPN (Table 1). At 30 h postinfection, cecal tissue RNA was harvested for qRT-PCR analysis on selected genes. A heat map shows the normalized fold change in expression values in log_2_ scale from two independent experiments in comparison to naive animals (*n* = 3 mice/experimental group). (B and C) qRT-PCR analysis of the indicated genes from cecal tissue RNA isolated from CDI and GF murine models at 30 h postinfection. Expression levels are shown relative to the CDI model PBS-treated group. Data are the mean ± SE (*n *= 3 to 6 mice/group). *, *P < *0.05; ***, *P < *0.001, between indicated groups.

With AXPN being the most efficacious drug in terms of animal survival and showing the greatest differences in gene expression in comparison to PBS-treated mice (Fig. 1B and 4C), we focused our efforts on further elucidating this drug’s mechanism of action in experimental CDI. By qRT-PCR analysis, we confirmed that AXPN significantly enhanced mRNA expression of several genes involved in immune protection (*Il33*, *Il22*, *Reg3b*, *Reg3g*) and recruitment of neutrophils (*Cxcl1* and *Cxcl2*) (Fig. 5A). Importantly, mRNA expression of several proinflammatory cytokines previously reported to adversely affect CDI outcome (*Il1b*, *Il6*, *Il23*, and *Tnfa*) (31–33) were downregulated in AXPN-treated animals compared to PBS-treated animals (Fig. 5A). The response induced by AXPN was localized to the site of infection, as treatment did not alter gene expression in the mesenteric lymph nodes of mice (Fig. S2) and IL-33 could not be detected in serum by ELISA (data not shown).

10.1128/mBio.00053-20.2 FIG S2 AXPN treatment does not alter cytokine expression in the mesenteric lymph nodes. Antibiotic-pretreated mice were infected with C. difficile and treated with AXPN or the vehicle control PBS. Gene expression levels in the mesenteric lymph nodes were determined by qRT-PCR 30 h postinfection. Expression levels are shown relative to those for the PBS-treated group as the mean ± SE (*n *= 5 mice/group). Download FIG S2, TIF file, 0.2 MB. Copyright © 2020 Andersson et al. 2020 Andersson et al. This content is distributed under the terms of the Creative Commons Attribution 4.0 International license.

Given the tissue-specific nature of the response, we hypothesized that the microbiome was required to generate the response in colonized tissue. To test this notion, we compared mRNA expression profiles in CDI and GF mouse models (Fig. 1A and 3A, respectively) 30 h postinfection. As seen in Fig. 5B and C, the AXPN-mediated response was significantly altered in GF mice. In the absence of the microbiota, upregulation of mRNA expression for *Il33*, *Il22*, and *Reg3g* by AXPN treatment was not evident, whereas expression of *Il1b*, *Il6*, and *Tnfa* was promoted and no different between treatment groups (Fig. 5B and C). Taken together, these data reveal that AXPN enhanced early antibacterial defenses during lethal CDI and required microbiome-derived factors to elicit protection.

### IL-33 is required for AXPN-mediated protection against lethal CDI.

**FIG 6:**
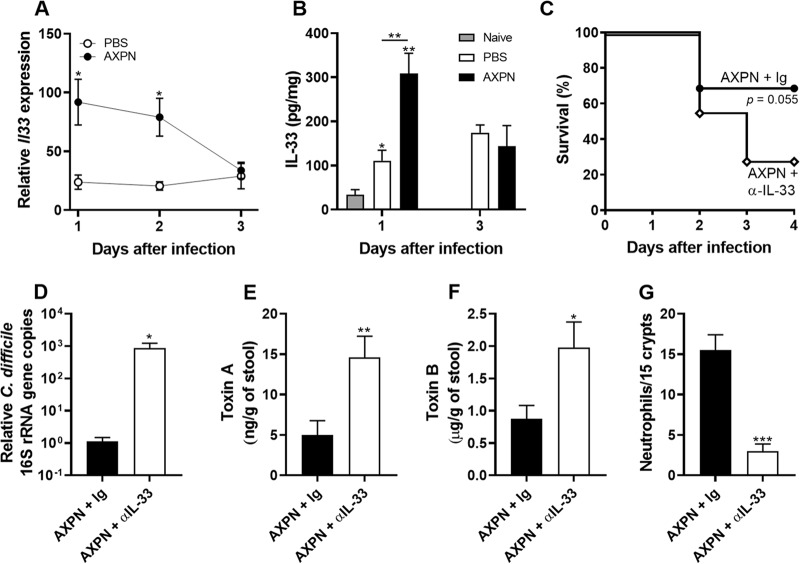
Upregulation of IL-33 occurs early and is necessary for AXPN-mediated protection. (A) qRT-PCR analysis of *Il33* mRNA in the ceca of antibiotic-pretreated mice infected with C. difficile spores and treated with AXPN or the vehicle control PBS. Data are shown relative to those for untreated, naive mice as the mean ± SE (*n *= 4 or 5 mice/group). *, *P < *0.05, between PBS- and AXPN-treated groups. (B) Levels of IL-33 protein assessed by ELISA (mean ± SEM, *n *= 4 or 5 mice/group). *, *P < *0.05; **, *P < *0.01, compared to results for naive or indicated groups. (C to G) Mice were treated with anti-IL-33 or Ig control antibodies 1 day prior to infection with C. difficile spores and drug treatment and every 48 h thereafter for up to 4 days. (C) Survival was monitored over the course of infection. (D) Bacterial burden in the cecal contents was determined at the time of necropsy by qPCR analysis of the quantities of 16S rRNA gene copies of C. difficile relative to the total number of 16S rRNA gene copies in the cecal contents. Expression levels are shown relative to that of the AXPN + Ig group on a log_10_ scale. (E and F) Toxin levels in cecal contents were determined by ELISA at 30 h postinfection. (G) Neutrophils in the lamina propria were quantified morphometrically. Data are the mean ± SE (*n *= 4 or 5 mice/group from two or three independent experiments). *, *P < *0.05; **, *P < *0.01; ***, *P < *0.001, compared to results for the AXPN + Ig or indicated groups.

IL-33 was recently shown to play an important role in host defense against CDI (34). With our finding that this cytokine represents a candidate upstream effector of drug activity (Fig. 4E), we next set out to determine whether IL-33 was involved in AXPN-mediated protection. We first examined cytokine expression at different time points early during infection and drug administration. In PBS-treated mice, *Il33* mRNA expression was upregulated (24-fold increase) 1 day postinfection and remained elevated through day 3 (Fig. 6A). However, administration of AXPN markedly augmented this response, with a maximum >90-fold induction at 1 day postinfection, followed by a gradual reduction to levels observed in PBS-treated controls (Fig. 6A). A similar effect was observed at the protein level, with AXPN-treated mice exhibiting significantly higher levels of IL-33 than PBS controls 1 day postinfection (Fig. 6B).

To determine the importance of IL-33 in drug protection against lethal CDI, AXPN-treated mice were administered either anti-IL-33 neutralizing antibodies or control antibodies. Blocking IL-33 by the neutralizing anti-IL-33 antibody during AXPN treatment diminished survival rates (Fig. 6C) and significantly increased C. difficile burden (Fig. 6D), with a corresponding elevation in toxin levels (Fig. 6E and F). Interestingly, anti-IL-33 antibody treatment also had a significant impact on the recruitment of neutrophils. Histological analysis revealed fewer neutrophils in the intestinal lamina propria of mice treated with both anti-IL-33 antibodies and AXPN than in mice treated with control antibodies and AXPN (Fig. 6G). Collectively, these results point to IL-33 being a key effector involved in AXPN efficacy, possibly through clearance of the pathogen by enhancement of neutrophil recruitment.

### Neutrophils are essential for AXPN-mediated clearance of C. difficile and IL-33 induction.

**FIG 7:**
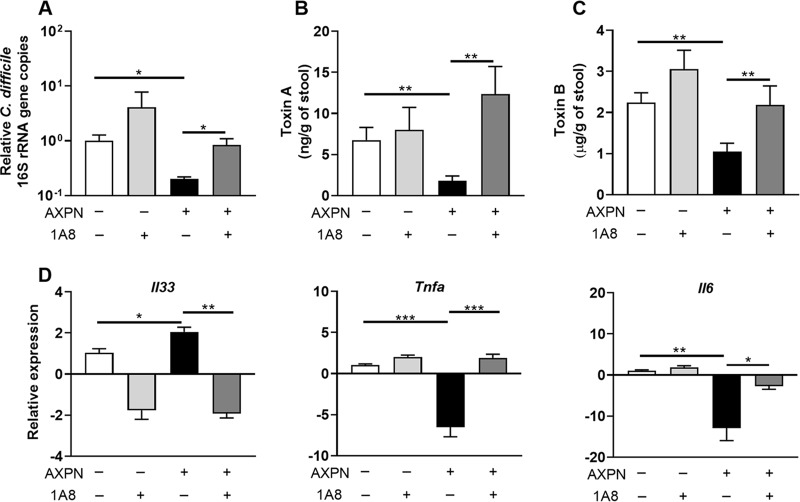
Neutrophils are essential for AXPN-mediated clearance of C. difficile. By use of the CDI model, anti-Ly6G, clone 1A8 antibody, or isotype control IgG was administered to mice 1 day prior to infection with C. difficile spores and treatment with AXPN or PBS. The antibodies were administered every 48 h for 4 days. (A) Thirty hours postinfection, the cecal contents were assessed for bacterial burden by qPCR analysis of the quantities of 16S rRNA gene copies of C. difficile relative to the total number of 16S rRNA gene copies in the cecal contents. Expression levels are shown relative to that of the PBS-treated group on a log_10_ scale. (B and C) Toxin levels in the cecal contents were also assessed 30 h postinfection by ELISA. (D) Cecal tissues were harvested 30 h postinfection, and the relative changes in mRNA levels of the indicated genes were measured using qRT-PCR. Expression levels are shown relative to that of the PBS-treated group. Data are the mean ± SE (*n *= 3 to 5 mice/group) and are representative of results from two independent experiments. *, *P < *0.05; **, *P < *0.01; ***, *P < *0.001, compared to results for the indicated groups.

While neutrophilic inflammation is a hallmark of C. difficile*-*associated disease (35), depletion of neutrophils markedly increases mortality in infection models (36, 37). Given that increased neutrophil counts were observed following AXPN administration and diminished with anti-IL-33 antibody treatment (Fig. 6G), we assessed the role of neutrophils in AXPN efficacy. Mice undergoing antibiotic pretreatment (Fig. 1A) were depleted of neutrophils through administration of a Ly6G-specific antibody, clone 1A8, which selectively depletes neutrophils while preserving nonneutrophil Gr-1^+^ cell populations, including macrophages (38). Light microscopic examination of intestinal tissues obtained at necropsy confirmed that administration of 1A8 antibody resulted in an 80% reduction in neutrophils compared to that of the isotype control group (data not shown), which was sufficient to diminish protection afforded by AXPN. In PBS-treated mice, depletion of neutrophils resulted in a modest increase in C. difficile burden and toxin titers (Fig. 7A to C). Surprisingly, AXPN treatment did not negate the effects of neutrophil depletion, with C. difficile burden and toxin titers being equivalent to or greater than those observed in PBS-treated controls (Fig. 7A to C), suggesting a decrease in the severity of CDI. In the absence of neutrophils, AXPN failed to induce *Il33* expression or significantly reduced expression of proinflammatory cytokine genes *Tnfa* and *Il6* (Fig. 7D), suggesting that AXPN protection against lethal CDI is dependent on the action of neutrophils to promote IL-33 production.

## DISCUSSION

Our studies demonstrate the potential of AXPN, DXP, and TFP as stand-alone therapeutics in alleviating the effects of CDI. Although these drugs belong to diverse pharmacological classes, all were able to reduce C. difficile burden and toxin levels through modulation of host innate immune defenses. Investigation into the mechanisms of AXPN revealed that drug administration was associated with increased neutrophil recruitment that contributed to an elevated IL-33 response, which was critical for pathogen control. Our findings paralleled recent observations that several FDA-approved drugs, including auranofin, an antirheumatic, and mistroprostol, a prostaglandin analog, were effective against experimental CDI (39–41). However, important differences in the modes of action exist between these drugs. Auranofin displays direct anti-C. difficile activity (40), which could lead to the development of resistance by the pathogen. Mistroprostol promotes recovery of the gut microbiome following antibiotic treatment; however, it is unclear whether the drug reduces C. difficile spore shedding, thereby lowering the risk for disease recurrence (41). In contrast, we showed that AXPN, DXP, and TFP acted as host-directed therapeutics in promoting immune responses that reduced the extent of infection. Although pathogens are less prone to develop resistance to host-directed therapeutics (42, 43), most are not considered stand-alone therapies and are often combined with antimicrobial agents as adjunct therapy. Indeed, our previous report showed that when combined with a subclinical dose of vancomycin, AXPN, DXP, and TFP elicited enhanced protection of mice against CDI, with 80 to 100% survival rates reported when treatment ensued 24 h postinfection. (23). Hence, our data demonstrate the potential utility of repurposing AXP, DXP, and TFP to combat CDI in high-risk patients and provide further evidence of the crucial involvement of the innate immune response in influencing disease outcome. Further studies optimizing the drug dosing regimen in mice would reveal whether they can be used as stand-alone therapeutics to achieve 100% protection against CDI. Developing and testing new treatment modalities against CDI have become worldwide priorities because of the high rates of disease incidence, recurrence, and mortality and the limited treatment options. Therapeutics that are less amenable to develop drug resistance and/or alter the gut microbiota, such as our lead drugs, are preferred choices and have the potential to provide a new approach to combatting CDI.

In elucidating the potential mechanisms of protection of these drugs, we observed reductions of both the C. difficile burden and toxin production in our experimental CDI model. However, as was reported with Y. pestis (22), none of the drugs had any direct effect on pathogen growth. With MICs determined to be >100 μg/ml, it is unlikely that AXPN, DXP, or TFP acts as a direct antimicrobial against C. difficile, as most breakpoints for antibiotic susceptibility fall below ≤16 μg/ml, based on both Clinical and Laboratory Standards Institute (CLSI) and European Committee on Antimicrobial Susceptibility Testing (EUCAST) guidelines (44, 45). It is also unlikely that these drugs directly target toxin A or B, as we demonstrated no effects on toxin production *in vitro* or *in vivo* in GF mice. Finally, it is unlikely that drug efficacy is the result of antimicrobial activities of metabolites, as neither of AXPN’s two metabolites, 7- and 8-hydroxyamoxapine, exhibited *in vitro* activity against the pathogen (data not shown).

The intestinal microbiome has a significant influence on CDI susceptibility and disease outcome and plays a key role in shaping the development of innate and adaptive immune responses. In agreement with previous reports (28, 29), we observed reduced diversity and shifts in microbiome composition in mice following antibiotic pretreatment and CDI. Although there was a noted reduction in *Peptostreptococcaceae* in drug treatment groups, which corresponds to the reduced bacterial loads of C. difficile in this experimental model, microbiome diversity and composition overall were unaffected by drug treatment. Remarkably, drug treatment also did not alleviate CDI in GF mice, with all animals succumbing to infection. Closer examination of immune responses following CDI and AXPN treatment revealed striking differences in gene expression profiles between CDI and GF murine models, with induction of genes encoding IL-33 and factors in the IL-22 pathway requiring the presence of the microbiota. These results are consistent with previous studies showing that GF mice have extensive immune deficits (46) and reduced expression of *Il33*, *Il22*, and *Reg3g*, which are associated with regulating intestinal inflammation (47–49). These findings suggest that the efficacy of these drugs is not a result of restoring the microbiota to a healthy state during early stages of infection. Additionally, the lack of efficacy of these drugs in GF animals further supports the conclusion that drug efficacy is reliant on host responses that develop in the presence of the microbiome.

Host responses to infection are increasingly being recognized to play a critical role in CDI outcomes (50). Studies in various knockout as well as aged mice have revealed a crucial role for early innate immune responses in protection against CDI, including neutrophil recruitment, generation of nitric oxide through *Nos2*, and production of IL-22 and its downstream antimicrobial proteins, including calprotectin proteins (*S100a8* and *S100a9*) and lipocalin-2 (*Lcn2*) (36, 37, 51–54). A recent study identified IL-33, an alarmin cytokine, as being protective against CDI, with prophylactic administration reducing mortality (34). Following AXPN treatment and CDI, we found dramatic upregulation of *Il33* gene expression and cytokine levels in the ceca of mice early during infection. This upregulation appears to be site specific, with no enhanced expression observed in mesenteric lymph nodes and IL-33 protein levels remaining below the limit of detection in the serum. Intriguingly, although Frisbee et al. concluded that IL-33-induced protection was related to the action of type 2 innate lymphoid cells (ILC2s), through enhanced levels of IL-5, IL-13, and eosinophilia (34), we observed no enhanced expression of these cytokines following drug treatment. Instead, our data suggest that AXPN-mediated protection results from activation of an alternate IL-33 pathway. Neutralization of IL-33 diminished AXPN efficacy and reduced neutrophil infiltration to the site of infection, indicating that IL-33 and neutrophil signaling pathways play a crucial role in this drug’s mechanism of protection. Whether this can be extrapolated to the protection mechanisms of DXP and TFP requires further investigation, although our RNA-seq data and IPA are supportive of this possibility.

Enhancement of neutrophil migration in response to IL-33 has been reported in several disease models (55–58). For instance, in Candida albicans infection, IL-33 pretreatment enhanced neutrophil killing activity, resulting in rapid fungal clearance and reduced mortality (58). In CDI, early neutrophil recruitment mediated by MyD88 and nucleotide-binding oligomerization domain 1 (NOD1) signaling have been reported to protect against lethality (36, 37). In *Nod1* knockout mice, reduced bacterial clearance and increases in indicators of systemic inflammation were noted, suggesting that loss of a specific local inflammatory response mediated by neutrophil infiltration resulted in more severe and uncontrolled inflammation (37). Moreover, depletion of neutrophils or chemokines involved in their recruitment, including CXCL1, markedly increases mortality in mice (36), providing further support of their role in protection against fulminant CDI. Yet, a hallmark of severe CDI in both mice and humans is marked neutrophil infiltration and subsequent inflammation (32, 35, 59), indicating that while a robust innate immune response is crucial to protection, this response must be tightly regulated to mitigate subsequent intestinal damage. In agreement with these studies, we demonstrated diminished efficacy of AXPN following the depletion of neutrophils, with increased C. difficile and toxin burdens and altered expression of genes encoding the innate immune response factors IL-33, IL-6, and tumor necrosis factor alpha (TNF-α). How AXPN upregulates IL-33 expression remains to be determined, but our results indicate that AXPN-induced IL-33 resulted in enhanced neutrophil infiltration to the site of infection, ultimately leading to improved bacterial killing and early resolution of inflammation. Finally, our lead drugs had little to no effect on the microbiota but did require the microbiota for induction of IL-33. Identification of the microbial protective communities involved in this process requires transgenerational studies to reconstitute an intact immune system in mice, which will be addressed in our future studies.

In summary, this study extends our previous reports of AXPN, DXP, and TFP as broadly protective against both Gram-negative and Gram-positive bacterial pathogens. This work also advances our understanding of host immune responses to C. difficile, demonstrating a potential role for IL-33-responsive neutrophils in pathogen clearance and toxin reduction. Although these drugs are not in the same class, all three target the central nervous system. Whether all three drugs share protective mechanisms, possibly through common neuro-immune signaling, remains to be determined and warrants future investigations. Finally, in our study, clinical efficacy of the drugs was achieved in mice using doses that are lower than human therapeutic doses. This is important, as it signifies that allometrically scaled doses in humans will be effective and will limit potential side effects of the drugs.

## MATERIALS AND METHODS

### Mice.

C57BL/6 mice were purchased from the Jackson Laboratory and housed under specific-pathogen-free conditions until experimental infection or were bred in-house. Germfree (GF) C57BL/6 mice were bred at Baylor College of Medicine (BCM) and maintained in flexible-film isolators (Class Biologically Clean) until use. All animal studies were approved by the Institutional Animal Care and Use Committees of the University of Texas Medical Branch (UTMB) and BCM.

### Bacterial infections.

C. difficile strain VPI 10463 (ATCC 43255) was grown anaerobically at 37°C in cooked meat medium (Fluka) or brain heart infusion medium (Difco) supplemented with yeast extract (BHIS), as previously described (60, 61). Spores were freshly isolated and resuspended in water until use (61). Adult C57BL/6 mice (>7 weeks old) of both sexes were administered an antibiotic cocktail consisting of colistin (850 U/ml; Sigma-Aldrich), gentamicin (0.035 mg/ml; Aspen), kanamycin (0.4 mg/ml; Sigma-Aldrich), metronidazole (0.215 mg/ml; Hospira), and vancomycin (0.045 mg/ml; Hospira) in the drinking water for 3 to 4 days and then switched to regular water. Animals then received clindamycin (32 mg/kg; Alvogen) via intraperitoneal (i.p.) injection for up to 2 days at 24-h intervals (60). Following antibiotic pretreatment, mice were infected by oral gavage with 10^5^ C. difficile spores (CDI model) (Fig. 1A). For GF mice, antibiotics were not administered, and animals were infected by oral gavage with 10^4^ spores (Fig. 3A). Concurrently with infection, animals were divided into groups and dosed with TFP (1.5 mg/kg in PBS; Sigma-Aldrich), AXPN (3 mg/kg in PBS; Sigma-Aldrich), or DXP (20 mg/kg in PBS; obtained from the UTMB pharmacy) by the i.p. route. TFP was given as a single dose, whereas AXPN and DXP were given every 24 h for 3 days (22). All animals were monitored twice daily for signs of infection, including weight loss, dehydration, and hypothermia, and assessed for clinical scoring parameters, including body condition, coat appearance, diarrhea, natural activity, and provoked activity levels (Fig. 1A and 3A). All parameters were scored between 0 and 3, with cumulative scores between 0 to 15.

### DNA extraction.

Total genomic DNA was extracted from mouse cecal content samples using methods previously described (62, 63). Briefly, samples were homogenized in lysis buffer and centrifuged, and the supernatants were subjected to precipitation with ammonium acetate, followed by an additional purification step with isopropanol (50% [vol/vol]) at –20°C. DNA samples were washed and then purified using a DNA Clean and Concentrator kit (Zymo Research).

### Quantification of C. difficile and toxins.

Quantitative PCR (qPCR) was used to determine the quantity of C. difficile 16S rRNA gene copies relative to the total quantity of bacterial 16S rRNA gene copies, as previously described (64), in mouse cecal content samples. Template DNA was extracted as described above, and 5 ng was used for qPCR with SYBR green PCR master mix (Applied Biosystems) in a CFX96 Touch real-time PCR detection system (Bio-Rad). The primers used are described in Table S2 in the supplemental material. Samples were assayed in duplicate and averaged. The relative amount of C. difficile 16S rRNA was calculated by the 2^−ΔΔ^*^CT^* threshold cycle (*C_T_*) method (64, 65). Levels of C. difficile toxins A and B were measured by ELISA using the C. difficile toxin A or B quantikit (tgcBiomics). Samples were assayed in duplicate, and the averages were used to calculate the mean ± standard error of the mean (SEM) per group.

10.1128/mBio.00053-20.4 TABLE S2 Primers used in this study. Download Table S2, DOCX file, 0.01 MB. Copyright © 2020 Andersson et al. 2020 Andersson et al. This content is distributed under the terms of the Creative Commons Attribution 4.0 International license.

### *In vitro* anti-C. difficile assays.

A starter culture of C. difficile VPI 10463 was prepared by inoculating a single C. difficile colony grown on BHIS agar into BHIS broth for overnight growth at 37°C under anaerobic conditions. The culture was reinoculated into 500 μl of fresh BHIS medium containing a 33 μM concentration of AXPN, DXP, TFP, or vancomycin or PBS to achieve an optical density at 600 nm (OD_600_) of 0.1. The cultures were incubated under anaerobic conditions at 37°C, with samples taken at designated time points for OD_600_ measurement. For MIC determinations, the broth microdilution method was used, with 100 μg/ml being the highest concentration tested.

### 16S rRNA gene sequencing and microbiome analysis.

Extracted microbial DNA from mouse cecal contents underwent amplification and sequencing of the V4 region of the 16S rRNA gene using a NEXTflex 16S V4 Amplicon Seq kit 2.0 (PerkinElmer), and sequences were generated on the Illumina MiSeq platform (Illumina). Raw reads were filtered using the Lotus pipeline (66), followed by *de novo* clustering to operational taxonomic units (OTUs) at 97% sequence identity with UPARSE (67). Bacterial diversity and community composition were evaluated using QIIME v1.8 (68), and taxonomy assignment of the representative sequence for each OTU was completed using the RDP classifier algorithm and the SILVA reference database (v123) (69).

### RNA isolation and analysis.

Whole mouse tissue samples were treated with RNAlater (Ambion) at 4°C for 24 h, snap frozen, and stored at –80°C until use. Total RNA was isolated using a Directzol kit (Zymo Research), and the quantity and quality were assessed using a NanoDrop spectrophotometer (Thermo Fisher Scientific). RNA-seq on total RNA isolated from cecal tissues was performed by LC Sciences. Briefly, poly(A) enrichment was used to perform ribosomal reduction on 2 μg total RNA. Following purification, the mRNA was reverse transcribed into cDNA. Single-end sequencing was performed on an Illumina HiSeq 2500. For analysis, raw reads (fastq files) were uploaded to the Partek Flow server (Partek). Sequences were aligned to the mm10 assembly of the mouse genome using STAR 2.5.3a, and the resulting summary of reads was quantified at the gene level to Ensemble transcripts 83 using Partek’s expectation maximization (E/M) annotation model. Gene counts were normalized to total read counts per sample and then log transformed (with an offset of 0.0001). To identify differentially expressed genes, statistical analysis was performed using Partek’s gene-specific analysis (GSA) multimodel estimation algorithm. Hierarchical clustering was performed on normalized and log-transformed counts with Partek Genomics Suite 6.6 using average linkage and Euclidian distance. Enriched pathways and upstream targets of the top upregulated transcripts (>4-fold altered expression) common between drug treatment groups were generated using IPA (Qiagen). Heat maps of select genes were generated by loading log_2_-transformed fold changes onto Morpheus software (https://software.broadinstitute.org/morpheus/).

Quantitative real-time PCR (qRT-PCR) was used to further measure mRNA expression as well as confirm selected RNA-seq data. Following isolation from the indicated tissues, total RNA was reverse transcribed into cDNA using iScript reverse transcription supermix (Bio-Rad). The cDNA was then used to perform qRT-PCR, using iTaq universal SYBR green supermix (Bio-Rad) on a CFX96 Touch real-time PCR detection system (Bio-Rad). Quantification of gene expression was performed using the 2^−ΔΔ^*^CT^* method with *Gapdh* as a reference gene (65, 70). Primers used are shown in Table S2.

### Quantitation of IL-33.

Cecal tissue samples were homogenized in PBS with 0.5% Triton-X and EDTA-free protease inhibitors (Roche) and centrifuged, and supernatant was frozen at –80°C. IL-33 levels were determined in the supernatants or serum by ELISA (R&D Systems) and normalized to total protein assessed by the Pierce bicinchoninic acid (BCA) protein assay (Thermo Fisher Scientific).

### Histological analysis.

Cecal tissues were harvested, opened longitudinally, and Swiss rolled prior to fixation for 48 h in 10% formalin. Following paraffin embedding, 5-μm sections were prepared and stained with hematoxylin and eosin (H&E). Neutrophils were identified by nuclear morphology at ×100 magnification and enumerated per 15 crypts in four noncontiguous areas. The scores were averaged and used to calculate the mean ± SEM for each group (71).

### *In vivo* IL-33 neutralization.

Anti-mouse IL-33 antibodies and its isotype control were purchased from R&D Systems. Mice undergoing antibiotic pretreatment for the CDI model (Fig. 1A) were injected i.p. with a dose of 5 μg of antibody or isotype control 1 day prior to infection and every 48 h thereafter over the course of the experiment, up to 4 days.

### *In vivo* depletion of neutrophils.

Mice were injected i.p. with 200 μg of anti-Ly6G, 1A8 clone (Bio X Cell) antibodies starting 1 day prior to infection and every 48 h thereafter over the course of the experiment, up to 4 days. Control mice were injected with an equivalent amount of the isotype control or PBS.

### Statistical analysis.

Survival curves were estimated using the Kaplan-Meier method, and differences between groups were tested using the log rank test. Significant differences in alpha and beta diversity were assessed using the Mann-Whitney U test. Other comparisons between groups were evaluated by Student's *t* test or one-way analysis of variance (ANOVA) as appropriate. Results were plotted in GraphPad Prism 8 (GraphPad Software), with results expressed as means ± SE. *P* values of <0.05 were considered significant.

### Data availability.

RNA-seq data were deposited in the GEO database at the NCBI (accession number GSE144291). The RNA-seq data used for hierarchical clustering are provided in Table S1.
