# Supplementary figures and images for: New Host-Directed Therapeutics for the Treatment of Clostridioides difficile Infection
Source: mBio. 2020 Mar 10;11(2):e00053-20. doi: 10.1128/mBio.00053-20 (PMC7064747; doi:10.1128/mBio.00053-20)

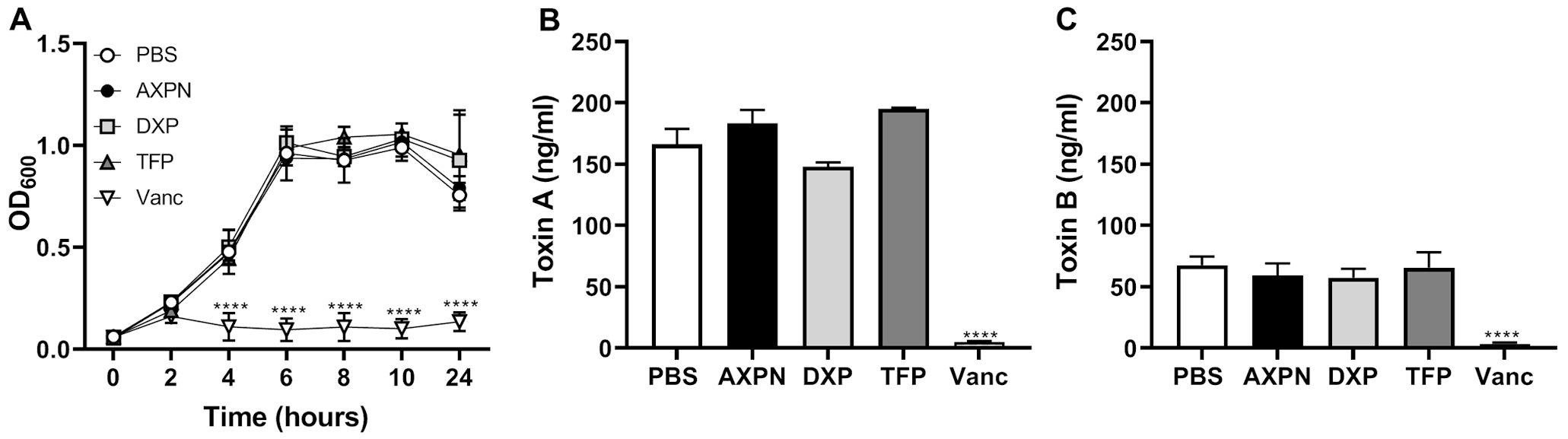

Supplement: FIG S1 [file mBio.00053-20-sf001.tif]

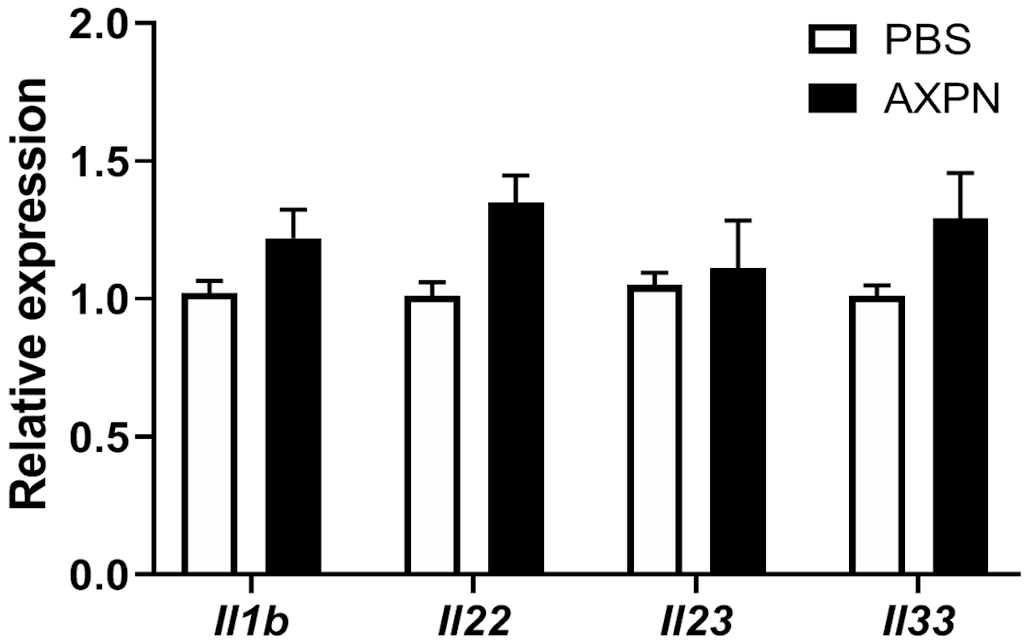

Supplement: FIG S2 [file mBio.00053-20-sf002.tif]
