# Supplementary material for: New Host-Directed Therapeutics for the Treatment of Clostridioides difficile Infection
Source: mBio. 2020 Mar 10;11(2):e00053-20. doi: 10.1128/mBio.00053-20 (PMC7064747; doi:10.1128/mBio.00053-20)
Supplement: TABLE S2 [file mBio.00053-20-st002.docx]

| **Table S2. Primers used in this study** | |
| --- | --- |
| **Gene** | **Primer sequences (5’ – 3’)** |
| *C. difficile 16s* | TTGAGCGATTTACTTCGGTAAAGA  CCATCCTGTACTGGCTCACCT |
| Total bacterial *16s* | GCAGGCCTAACACATGCAAGTC  CTGCTGCCTCCCGTAGGAGT |
| *Il1a* | ggcacggggactgccctctat  tgtcggggtggctccact |
| *Il1b* | TGCCACCTTTTGACAGGTCA  TCCCTGTAGTGACAGCACCT |
| *Il6* | AGACAAAGCCAGAGTCCTTCAGAGA  GCCACTCCTTCTGTGACTCCAGC |
| *Il18* | AGCAATCCTCCTGTCCCCGT  CAGGTCGTGGCCGAATCTCC |
| *Il22* | GCCAGCCTTGCAGATAACAAC  GCTGAGCTGATTGCTGAGTTTGGT |
| *Il23* | TGGCATCGAGAAACTGTGAGA  TCAGTTCGTATTGGTAGTCCTGTTA |
| *Il33* | AGAATGCCTGACATTGGTGC  ACACAGAGTCTTATTTGGTA |
| *Il36b* | acaaaaagcctttctgttctatcat  ccatgttggatttacttctcagact |
| *Il36g* | AGAGTAACCCCAGGTCAGTGG  GTGGGCCCCATTGAAAGTTA |
| *Cxcl1* | TGGCTGGGATTCACCTCAAGAACA  AGTGTTGTCAGAAGCCAGCGTTCA |
| *Cxcl2* | AACTGCGCTGTCAATGCCTGAAGA  TCCAGGTCAGTTAGCCTTGCCTTT |
| *Ccl2* | TTGTCACCAAGCTCAAGAGA  ATTAAGGCATCACAGTCCGAGT |
| *Ccl3* | TGAGAGTCTTGGAGGCAGCGA  TGGCTACTTGGCAGCAAACA |
| *Lcn2* | TTTCACCCGCTTTGCCAAGT  GTCTCTGCGCATCCCAGTCA |
| *Ly6g* | TTGTATTGGGGTCCCACCTG  CCAGAGCAACGCAAAATCCA |
| *Tnfa* | TTCCAGAACTCCAGGCGGTGC  TGAGTGTGAGGGTCTGGGCCAT |
| *Reg3α* | TGTGCATGATGTGCTTGCCT  GGCTTCCAGCACCCACATAG |
| *Reg3b* | TGGGAATGGAGTAACAATGA  GGCAACTTCACCTCACATGT |
| *Reg3g* | CCATCTTCACGTAGCAGCAG  CAAGATGTCCTGAGGGC |
| *S100a8* | tgccctctacaagaatgact  aagctctgctactccttgtg |
| *S100a9* | cgacaccttccatcaatact  tcagcatcatacactcctca |
| *Gapdh* | GTGCAGTGCCAGCCTCGTCC  GCCACTGCAAATGGCAGCCC |
